# Electrospinning of Poly-3-Hydroxybutyrate Fibers Loaded with Chlorophyll for Antibacterial Purposes

**DOI:** 10.3390/polym16223221

**Published:** 2024-11-20

**Authors:** Polina M. Tyubaeva, Ivetta A. Varyan, Roman R. Romanov, Vasily A. Merzlikin, Olga A. Gruznova, Dmitry V. Gruznov, Nikolay I. Popov, Gulizar Sh. Shcherbakova, Ekaterina N. Shuteeva, Irina P. Chesnokova, Anton V. Lobanov, Anatoly A. Olkhov

**Affiliations:** 1 Department of Physical Chemistry of Synthetic and Natural Polymer Compositions, Emanuel Institute of Biochemical Physics, Russian Academy of Sciences, 4 Kosygina St., 119334 Moscow, Russia; ivetta.varyan@yandex.ru (I.A.V.); aolkhov72@yandex.ru (A.A.O.); 2 Academic Department of Innovational Materials and Technologies Chemistry, Plekhanov Russian University of Economics, 36 Stremyanny Per., 117997 Moscow, Russia; otmetkin@mail.ru; 3 Laboratory of Liquid-Phase Oxidation, Semenov Federal Research Center for Chemical Physics, Russian Academy of Sciences, 4 Kosygina Street, 119334 Moscow, Russiachesnokkova-irishka@mail.ru (I.P.C.); 4 Laboratory of Veterinary Sanitation, All-Russian Research Institute of Veterinary Sanitation, Hygiene and Ecology—Branch of Federal State Budget Scientific Institution “Federal Scientific Center—K.I. Skryabin, Ya.R. Kovalenko All-Russian Research Institute of Experimental Veterinary Medicine, Russian Academy of Sciences”, 5 Zvenigorodskoye Highway, 123022 Moscow, Russiaekashyt@mail.ru (E.N.S.); 5 Laboratory of Veterinary Sanitation and Environmental Safety in Beekeeping, All-Russian Research Institute of Veterinary Sanitation, Hygiene and Ecology—Branch of Federal State Budget Scientific Institution “Federal Scientific Center—K.I. Skryabin, Ya.R. Kovalenko All-Russian Research Institute of Experimental Veterinary Medicine, Russian Academy of Sciences”, 5 Zvenigorodskoye Highway, 123022 Moscow, Russia; 6 Department of Chemistry and Technology of High-Molecular Compounds Named After S.S. Medvedev, MIREA—Russian Technological University, 78 Vernadsky Avenue, 119454 Moscow, Russia; 7 Department of General Chemistry, Moscow Pedagogical State University, 1/1 Malaya Pirogovskaya Street, 119435 Moscow, Russia

**Keywords:** poly-3-hydroxybutyrate, electrospinning, chlorophyll, antibacterial properties

## Abstract

This work is devoted to the creation of biocompatible fibrous materials with a high antimicrobial effect based on poly-3-hydroxybutyrate (PHB) and chlorophyll (Chl). The data obtained show the possibility of obtaining fibrous materials from PHB and Chl by electrospinning methods. The obtained electrospun matrices were investigated by the SEM, DSC and FTIR methods. Various key properties of the matrices were evaluated, including hydrophilicity and mechanical strength, as well as photodynamic and light-dependent antimicrobial effects against the conditionally pathogenic microorganism *Staphylococcus aureus*. The results demonstrate a significant improvement in electrospinning properties for a concentration of 0.5% Chl and a reduction in fiber formation defects, as well as an increase in the strength of nonwovens. It was found that the antimicrobial potential of Chl-PHB (with concentrations of Chl of 1.25 and 1.5%) is higher than that of Chl in free form. It was also determined that irradiation increases the inhibitory effect of Chl, both in free form and in the form of a complex with a polymer.

## 1. Introduction

The development of antimicrobial nonwoven materials attracts considerable interest nowadays [1]. Three main approaches are used to impart antibacterial properties such as antiadhesion, biocide release and contact-active antimicrobial modification [2]. 

The problem of microorganisms’ resistance to many existing antibiotics has become a pressing issue, irrespective of the method of modification, and a number of existing antimicrobial agents should be subjected to certain criticism. There has been some research on the toxicity of metal nanoparticles [3]. The mechanism of toxicity may be related either to the presence of free metal ions [4] or to the small size of nanoparticles, which allows them to penetrate easily into the body, bypassing protective barriers, the respiratory system and the digestive tract [5]. The well-known antimicrobial properties of silver nanoparticles show greater toxicity than macroparticles, due to the induction of oxidative stress, which leads to mitochondrial dysfunction and increased permeability of cell membranes [6]. Aluminum and copper oxides are also highly antimicrobial. They have been criticized for their negative effects on mitochondrial function and reduced cell viability [7]. As a result, the search for new antimicrobial agents of natural origin that are safe for living organisms is a major focus today.

Chlorophyll (Chl) is one of these promising compounds with high antimicrobial activity [8]. Chl is a key molecule in plant photosynthesis. It is widely known as a green pigment and a porphyrin, activating the energy transport process [9]. 

According to the study of I. Stojiljkovic et al., the mechanism of the light-independent inhibition of Chl and other metalloporphyrins consists in the fact that they enter into microbial cells through interaction with heme receptors and bind to cytochromes that interfere with electron transfer to oxygen and cause the generation of active oxygen species [10]. Furthermore, chlorophylls and their derivatives are well known as photosensitizers that can generate singlet O_2_ with sufficient quantum yields. This has led to their use in antimicrobial photodynamic therapy (PDT) applications [11]. 

Chlorophyll contains four pyrrole nitrogen rings bonded to a central magnesium atom and a fifth ring containing carbon atoms and a long phytol tail. The phytol tail of chlorophyll confers hydrophobicity and limits binding efficacy with respect to carcinogens and mutagens [12]. Therefore, the development of ways to control the hydrophilic properties of Chl is particularly important. There is also great interest in designing biomimetic polymer systems for targeted delivery and sustained release of Chl for controlled antimicrobial activity.

Despite the limitations due to the tendency of Chl to form aggregates as well as the problems of immobilizing Chl at room temperature, there have been numerous attempts to create a stable polymer–Chl system [13]. The electrospinning (ES) process has been very successful in incorporating natural pigments and tetrapyrroles into a polymer matrix [14,15]. 

Successful ES of polyacrylonitrile–Chl systems was demonstrated by Ince Yardimci et al. and Liu et al. [16,17]. Polylactide–Chl systems were successfully obtained by blotting of nonwoven materials in the work of Williams et al. [18]. However, these polymers have very high glass transition temperatures (above 70 °C for polyacrylonitrile and above 60 °C for polylactide), which makes it difficult to rapidly absorb and control the release of Chl, due to the glassy nature of the amorphous phase of the polymer matrix. Sandra et al. and Cao et al. have successfully demonstrated polyvinyl alcohol–Chl nonwoven systems [19]. However, polyvinyl alcohol is soluble in water, making it difficult to control the hydrophilic properties of the material, thus limiting its application [20]. The success of Jassin et al. in obtaining poly(methyl methacrylate)–Chl systems is particularly noteworthy [21]. Polymethylmethacrylate is an amorphous, rubber-like polymer. The choice of a biocompatible matrix for Chl with a glass transition temperature below room temperature, hydrolytic resistance, good forming properties for ES and the potential to create biomimetic structures is still a question. One such polymer is polyester of bacterial origin—poly-3-hydroxybutyrate (PHB). PHB is a thermoplastic, semi-crystalline polymer with a glass transition temperature of 6 °C which is hydrophobic and can be easily modified [22,23]. PHB can act as a matrix for antimicrobial agents and is characterized by sufficiently high strength properties and impressive biocompatibility [24,25,26]. 

In previous investigations, we studied the antimicrobial properties of Chl extracted from *Urtica dioica* in PLA and PVP polymer matrices against both Gram-positive and Gram-negative bacteria [27]. This article aimed to prepare antimicrobial matrices based on PHBs filled with different Chl concentrations by the ES method. In this work, we solved the problem of the preparation of molding solutions with different concentrations of Chl and studied the antimicrobial activity of the obtained materials against *Staphylococcus aureus*, a Gram-positive microorganism that colonizes mucous and cutaneous epithelia of animals and humans, which (due to decreased resistance of the host organisms) can show pathogenic characteristics. 

## 2. Materials and Methods

### 2.1. Materials

Poly-3-hydroxybutyrate (PHB) powder (16F, BIOMER, Frankfurt, Germany) with a molecular weight of 350 kDa and a density of 1.248 g/cm^3^; chlorophyll (Chl) as a Chl*a* and Chl*b* mixture (3:1) extracted from dried nettle leaves (*Urtica dioica*) [28]; chloroform (CL) Amresco (Solon, OH, USA); dimethyl sulfoxide (DMSO) (99.5%, PanReac Applichem, Barcelona, Spain); sterile physiological solution (0.9% NaCl, Khimikom, Nizhny Novgorod, Russia); meat-peptone agar (MPA, Khimikom, Nizhny Novgorod, Russia); meat-peptone broth (MPB, Khimikom, Nizhny Novgorod, Russia); and the industry turbidity standard for determining total microorganism concentrations (BAK-10 kit, Ormet, Yekaterinburg, Russia) were used in this work. *Staphylococcus aureus* (strain 209-P) was provided from the cell culture collection of the All-Russian Research Institute of Veterinary Sanitation, Hygiene and Ecology.

### 2.2. Preparation of PHB-Chl Matrices

#### 2.2.1. Preparation of Forming Solutions

The pre-dried PHB was dissolved in CL at a concentration of 7% at 60 °C in a magnetic stirrer for 12 h until a homogeneous transparent solution was obtained. Chl was dissolved with CL (75 mg in 25 mL) at 25 °C, followed by stirring for 60 min. Next, a portion of the Chl solution was selected and injected into the PHB solution with constant stirring at 25 °C. The solutions were homogenized for 60 min.

The parameters of the PHB-Chl solutions for electrospinning are shown in Table 1. The viscosity of the PHB-Chl solutions was measured using the Brookfield Rotary Viscometer DV2TLV according to the ASTM D2983 [29] with a spindle LV-3 at 25 °C (for 100 mL of solution). The electrical conductivity of the PHB-Chl solutions was measured using the SanXin DDS-11C Laboratory Conductometer (SanXin Instrumentation, CaoHeJing, Shanghai, China) according to GOST 8.292-2013 [30] at 25 °C (for 25 mL of solution).

#### 2.2.2. Electrospinning of PHB-Chl Solutions

Electrospinning (ES) of PHB-Chl solutions with different concentrations of Chl was performed using an EFV-1 ES scale with a single capillary (Moscow, Russia). The characteristics of the ES were as follows: a protective chamber with temperature and humidity control (the temperature was 25 °C; the humidity was 38–40%); pressure control (0–10 kgf/cm^2^); stationary collector electrode (30 × 30 cm^2^); voltage control (16–20 kV); the distance between the electrodes was 200–220 mm. The flow rate of the molding solution was calculated as the ratio of the polymer solution consumed on the web to the molding time and is shown in Table 1.

### 2.3. Investigation of Electrospun PHB-Chl Matrices

#### 2.3.1. Microscopy

Microphotographs of the PHB-Chl fibrous materials were obtained by the Tescan VEGA3 scanning electron microscope (Tescan, Wurttemberg, Czech Republic) with the platinum layer and an accelerating voltage of 20 kV.

The morphology of the fibers was investigated by the Olympus Stream Basic software (Olympus, Tokyo, Japan) on the optical microscope the Olympus BX43 (Olympus, Japan, Tokyo). Average diameters and diameter distributions were determined in 10 areas of the materials using z-stacking on an area of 900 × 650 mkm of each sample according to the standard technique [31].

#### 2.3.2. Surface Density

The surface density of PHB-Chl fibrous materials was investigated using the analytical weighing machine the Balance XPR106DUHQ/A (Mettler Toledo, Columbus, OH, USA) according to the standard technique [32]. Average values were counted from 10 iterations.

#### 2.3.3. Mechanical Properties

Mechanical properties were investigated by the universal testing machine the Instron electropuls e3000 (Instron, Norwood, MA, USA) with a load cell of 5 N capacity and a crosshead speed of 5 mm/min, according to a standard technique [33]. The room conditions were controlled at 22 °C and 40% relative humidity. Elongation at break and maximal strength were registered automatically. Average values were counted from 10 iterations. 

#### 2.3.4. DSC

The crystalline structure of PHB in the PHB-Chl matrices (melting temperature and enthalpy of melting) was investigated by the Netzsch 214 Polyma (Netzsch, Selb, Germany) in an argon atmosphere according to the standard technique [34]. The heating and cooling rate was 10 °K/min. Test samples were cut from 3 different areas of the electrospun material with a total weight of 7 mg. The enthalpy of melting and the melting temperature were calculated using Netzsch Proteus software. 

#### 2.3.5. FTIR

Infrared spectra were obtained by Fourier Transform IR (FTIR) spectroscopy using the Bruker LUMOS II Research Infrared Fourier Microscope (Bruker, Karlsruhe, Germany) with the module for measuring multiple disturbed total internal reflection on diamond crystal [35]. The range of measurement was 600–4000 cm^−1^, and the resolution was 2 cm^−1^. All spectra were taken at least 10 times in 3 areas of each sample and average values were calculated. Spectra of pure Chl were obtained by the KBr pellet technique, using 1 mg of powder in 50 mg of spectroscopic-grade KBr.

#### 2.3.6. Contact Angle

Wettability was investigated by contact wetting angle measurements on the FMA050 optical microscope with the Altami studio 3.4 software [36]. Water droplets (2 μL) were measured 5 times in 3 areas of each sample. The test samples were 30 × 30 mm^2^.

#### 2.3.7. Swelling

Water uptake ability was investigated by swelling tests performed according to a published technique [37]. The test samples were 10 × 10 mm^2^. The samples were weighed using VL-64 analytical scales (Gosmetr, Moscow, Russia) after different exposure times in phosphate-buffered saline (PBS). The measurements were carried out 3 times. The degree of swelling was calculated using the following formula:

(1) $$\frac{\mathrm{real}\;\mathrm{time}\;\mathrm{wet}\;\mathrm{weight}\;-\;\mathrm{dry}\;\mathrm{weight}\;}{\mathrm{dry}\;\mathrm{weight}\;}\times 100\%$$

### 2.4. Microbiological Assay

#### 2.4.1. Cells and Culture Conditions

The daily cultures of *Staphylococcus aureus* (209-P strain) were cultivated on a slanted MPA at a temperature of 37 °C for at least 24 h in a dry-air thermostat. Suspensions of 10^9^ colony-forming units (CFU) per milliliter were prepared from the daily cultures in sterile saline solution according to the turbidity standard. The obtained concentrations were confirmed using a spectrophotometer at λ = 600 nm. Then, serial dilutions with 10-fold increments (10^8^, 10^7^, 10^6^ and 10^5^ CFU/mL) were prepared from the suspensions of the daily cultures by titration in sterile saline solution.

#### 2.4.2. Minimum Inhibitory Concentration (MIC) Analysis

To determine the minimum inhibitory concentration (MIC) of the Chl and Chl-PHB, fragments of polymer forms with active substance contents of 3.75 to 90 μg and Chl solutions with equivalent active substance contents were prepared. The dilution step was 3.75 μg. Each sample was placed in a test tube containing sterile liquid nutrient medium (MPB) contaminated with *Staphylococcus aureus*.

Irradiation of the samples was performed in quartz cuvettes using a UV lamp (20 W) with a light irradiation range of 400–500 nm (emission peak—450 nm) for 25 min. Samples that were intended for studying the light-independent effect were not irradiated.

Next, the tubes were then incubated in a thermostat at 37 °C for 5 days. Observations were made daily to assess the presence of visible turbidity in the medium, which indicated bacterial growth. These results were compared with those obtained from control samples containing sterile medium and control samples not treated with the active substance or its polymeric form.

The experiments were carried out in triplicate.

#### 2.4.3. Reduction in Microorganism Growth Study

To calculate the percentage reduction in microbial cell growth in the presence of the tested preparations with irradiation and without it, the absorbance of a pure sterile medium MPB (sterility control) and a control inoculation with microorganisms without the addition of any of the preparations were measured, as well as sterile media with the addition of the preparations and experimental inoculation of microorganisms with the prepared solutions. The reduction in microbial growth in the presence of the tested preparations was calculated using the following formula:

(2) $$\left(1-\frac{\mathrm{absorbance}\;\mathrm{of}\;\mathrm{test}\;\mathrm{solution}\;-\;\mathrm{absorbance}\;\mathrm{of}\;\mathrm{corresponding}\;\mathrm{control}\;}{\mathrm{absorbance}\;\mathrm{of}\;\mathrm{assay}\;\mathrm{growth}\;\mathrm{control}\;-\;\mathrm{absorbance}\;\mathrm{of}\;\mathrm{sterility}\;\mathrm{control}\;}\right)\times 100$$

#### 2.4.4. Determination of Inhibition Zone Diameter

Determination of the antibacterial activity of Chl and Chl-PHB on a solid nutrient medium was performed by measuring the diameters of inhibition zones. To this end, a microbial suspension at a concentration of 10^5^ CFU/mL was inoculated in Petri dishes containing sterile MPA medium. From samples of the polymer matrices (preparation contents—0.5%, 0.75%, 1.25% and 1.5% (wt.%) relative to the mass of PHB), fragments with an area of 1 cm^2^ were placed in the center of the inoculated Petri dishes. Then, 100 µL of Chl solution containing an equivalent amount of active substance was added to the wells created with a sterile punch in the center of the MPA plates.

To study the photodynamic activity of the preparations, some of the Petri dishes with samples were irradiated with a UV lamp (20 W) with a light irradiation range of 400–500 nm (emission peak—450 nm) for 25 min. Samples that were intended for studying the light-independent effect were not irradiated. Samples intended for the detection of the light-independent effect were not irradiated.

Next, the samples were incubated in a thermostat (37 °C, 72 h). The results were recorded daily according to the diameter of the growth inhibition zone (mm). To confirm the lack of polymer toxicity, PHB samples without active substances were used. The experiments were carried out in triplicate.

To avoid the impact of light on the Chl, all the experiments were performed in a room with diffuse side lighting and without additional light sources.

## 3. Results

### 3.1. Structure and Properties of the PHB-Chl Matrices

**Figure 1:**
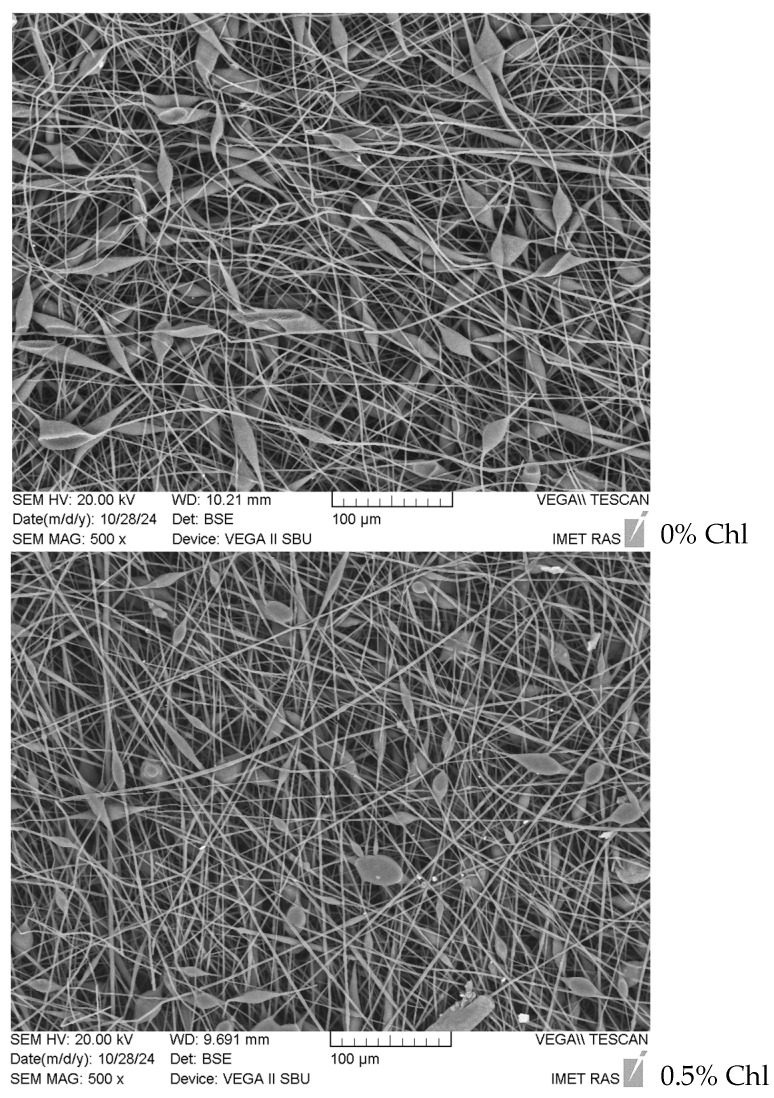

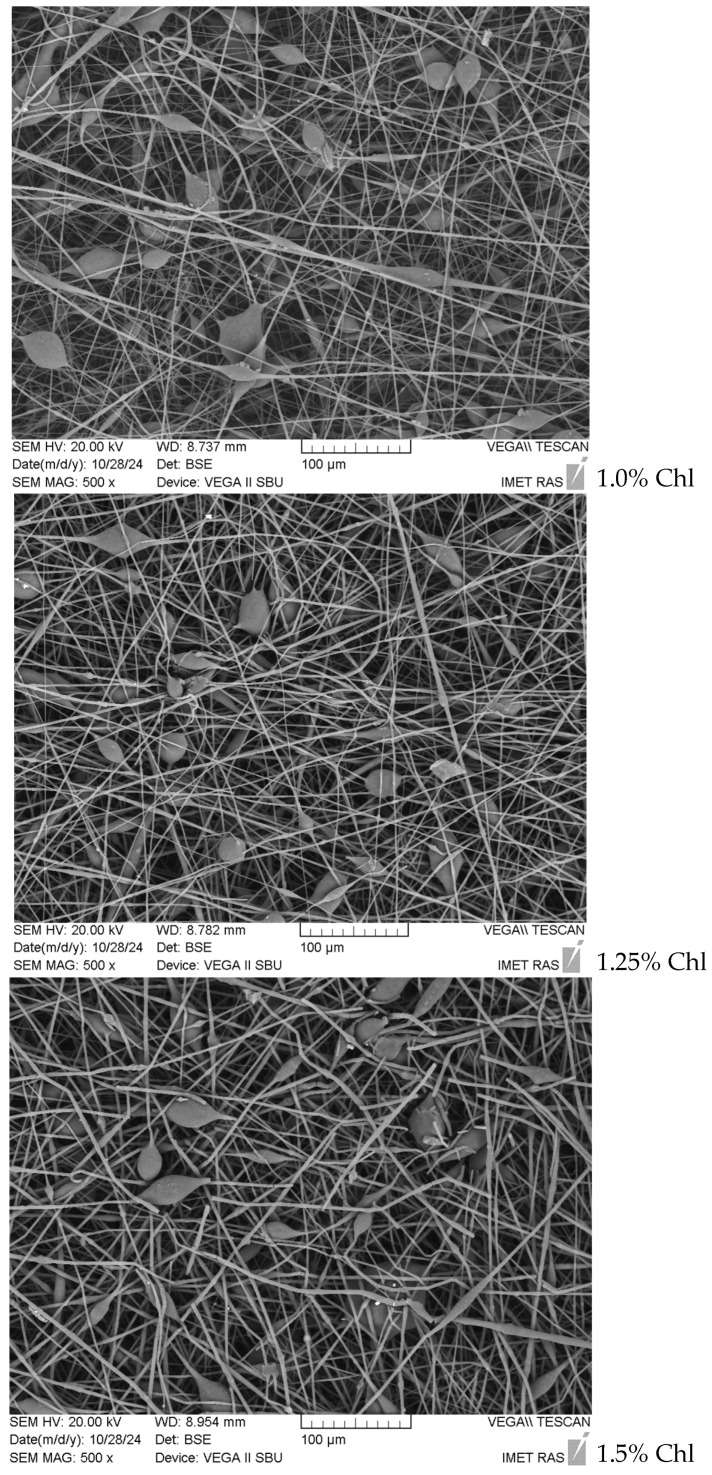
SEM microphotographs of PHB-Chl electrospun materials.

**Figure 2:**
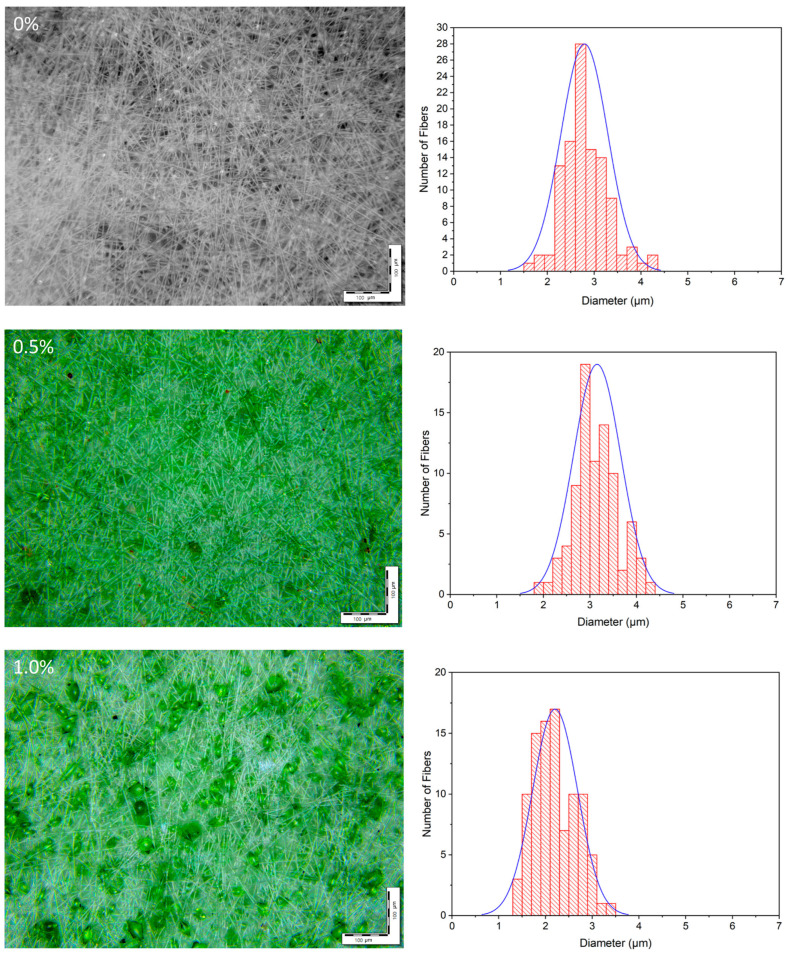

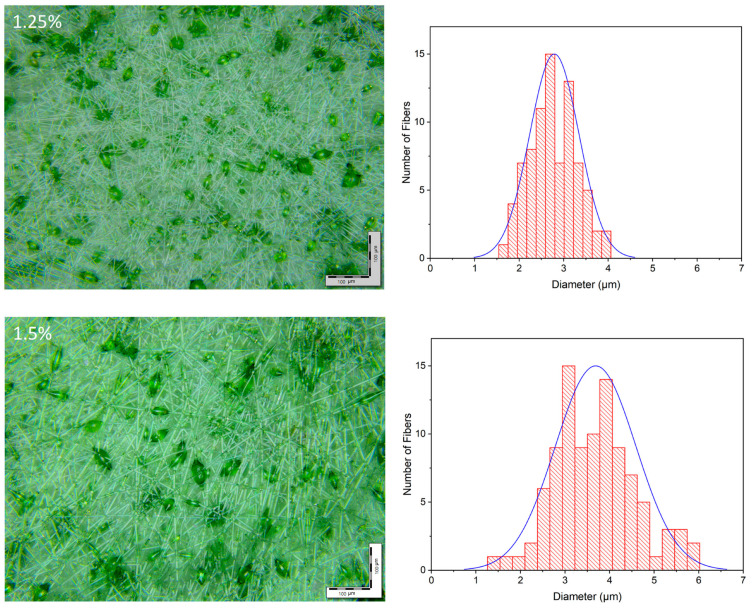
Microphotographs and average diameter distributions of PHB-Chl electrospun materials.

Microphotographs of the electrospun PHB-Chl matrices obtained by SEM are shown in Figure 1. Microphotographs obtained by optical microscopy in reflected light and the distributions of average diameters are shown in Figure 2. The results of the analysis of the morphology of the fibrous materials are presented in Table 2.

It is important to note that all the obtained materials had a highly developed surface and a high degree of porosity, which is typical for electrospun materials. The initial PHB fibers had many elliptical thickenings. The size of these thickenings varied from 20 to 35 μm. The origin of these defects may have been due to the low electrical conductivity of the ES solutions, leading to an irregular flow of electric charge through the primary jet of the polymer solution. As a result, an uneven deformation of the polymer in the jet could be observed. While this process occurs, competition arises between the orientation and relaxation processes of polymer macromolecules. Such thickenings are more common for lower-molecular-weight PHB. In PHB-CL systems, such fiber deformations are especially common when using PHB with a molecular weight of 300–500 kDa [38,39]. The limitation in the choice of PHB of a lower molecular weight is always due to its higher degradation rate and better biocompatibility in case of application in antimicrobial biomedical materials [40]. According to the study of Foster et al. [41], the formation of defects is not due to moisture. Thickening, according to the results presented in the studies of Thanh et al., Sadat-Shojai, Vanhausden et al. and Olkhov et al., can occur for a number of reasons: it may be due to the type of solvent, insufficient conductivity–viscosity balance in the system or the low molecular weight of the polymer [42,43,44,45]. As a consequence, a large number of approaches to their elimination are known, ranging from the variation of solvents, plasticizers and modifying additives to the introduction of more electrically conductive molecules, including polar ones [42,46,47]

Straight sections of PHB fibers are characterized by a cylindrical geometry and an average diameter of 2.7 μm, with a diameter range of 1.5 to 3.5 μm. 

With the addition of Chl, a decrease in the number of thickenings on the fibers could be observed, which, in our opinion, was due to the influence of polar chlorophyll molecules [48]. As shown in the works of Olkhov et al., Santos et al. and Li et al., the addition of polar molecules has an effect on fiber diameter, diameter distribution, electrospinning rate and even the thermal properties of fibers, due to the role of additives in the polymer crystallization process [48,49]. It should be noted that systems with polar substances are characterized by the presence of intermolecular interactions and the formation of more perfect crystalline fibrous structures [50].

**Figure 3:**
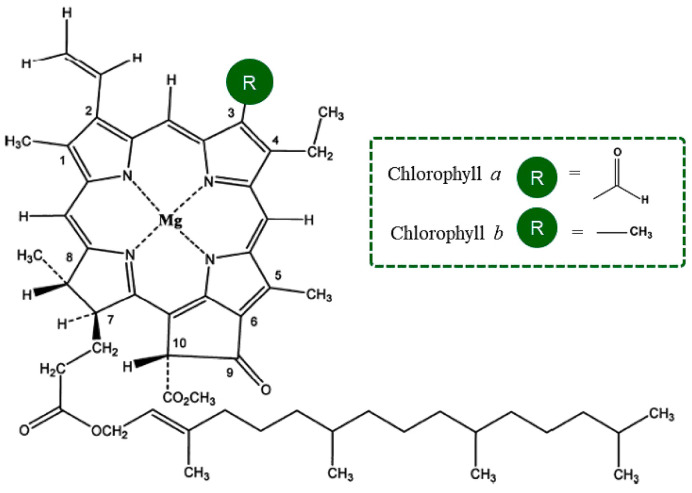
Molecular structures of chlorophyll a and chlorophyll b [51].

The structural formula of Chl is given in Figure 3.

**Figure 4:**
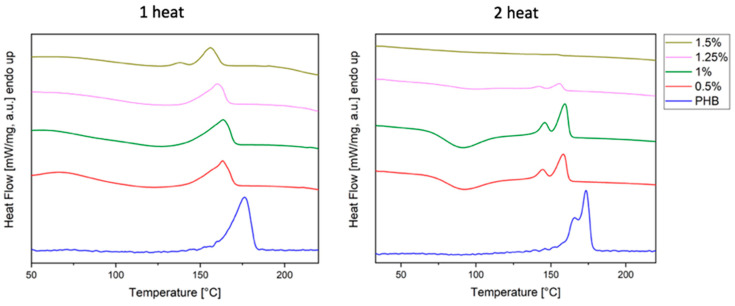
DSC curves of PHB-Chl electrospun materials.

Polar molecules of Chl equalize the electrostatic field at the moment of pulling a drop of ES solution into the interelectrode space during the ES process. It should be noted that with a content of 0.5% chlorophyll, thickenings were observed, the sizes of which were significantly lower than at a higher concentration (1–1.5%). Their number per unit area of the electrospun material was also significantly reduced. For 0.5% of Chl, the average number of thickenings was reduced by 64% in comparison to 1.5% of Chl. The increase in the size of the thickenings on the fibers was apparently due to the agglomeration of Chl molecules in the amorphous regions of the PHB fibers. And chlorophyll is characterized by a fairly high tendency to agglomeration [52]. From the data shown in Table 2, it can be seen that the average diameter of the PHB fibers practically does not depend on the concentration of Chl in the studied range. But it should be noted that at the maximum Chl content (1.5%), large-diameter fibers (5–6 μm) appeared, which was due to the effect of the additive on the viscosity of the polymer solution. The presence of fibers of various diameters, from 1.2 to 6 microns, in nonwoven fabric makes it possible to form a material with a denser structure, where fibers of small diameters fill the spaces between large fibers. This is indicated by the data on the surface density, which was at a maximum for nonwoven fibrous materials with a chlorophyll content of 1.5%. In addition, the introduction of chlorophyll makes it possible to form a thicker layer of material. To study the effect of Chl on the supramolecular structure of PHB in fibers, the DSC method was used. The results are shown in Table 3 and Figure 4.

It is important to note that PHB is a semi-crystalline polymer, the crystallization of which is initiated mainly by the homogeneous formation of crystallization nuclei, which can lead to a very low density of their formation [53]. This can be well observed from the DSC curve of pure PHB (1 heat, Figure 4). As a result, cold secondary crystallization occurs, which also affects the formation of the amorphous phase [54]. This can be observed from the DSC curve of pure PHB (2 heat, Figure 4), where approximately 40% of the crystal did not have time to crystallize under the experimental conditions and a differentiated low-temperature shoulder formed in the range of 154–160 °C. Secondary crystallization could also have led to a decrease in the mechanical properties of the polymeric material and to the noticeable differentiation of the melting peak in the DSC curves, where two types of crystalline formations can be observed: small (incomplete) fractions, which melt at lower temperatures, and large (completed) fractions, which melt in the range of the pure PHB melting region [55]. Many researchers note the role of nucleating particles and copolymers of various natures as nuclei of the crystallization process of PHB [56,57,58]. And such a role is quite likely for the chlorophyll molecule, given its structure. However, in practice, we see how aggregation prevails over the ability to nucleate PHB. As can be seen from Figure 4, it is impossible to talk about the nucleating effect of Chl in the PHB-Chl system. However, a significant effect on the crystallization of PHB can be observed. The melting point of chlorophyll a is 117–120 °C, and the melting point of chlorophyll b is 120 °C [59]. Thus, as the chlorophyll content increases, the crystallinity of PHB decreases by more than three times, which indicates an obstacle to the formation of a crystalline phase. The thermal properties of PHB in the PHB-Chl electrospun materials are shown in Table 3.

It can be seen that with an increase in the concentration of Chl, the heat and melting point of the crystalline phase of PHB decrease. The dependence persists even with the repeated melting of materials. With a Chl content of 1.5%, a low-temperature peak of PHB melting can be observed, which may indicate the incompleteness of the polymer crystallization process. During the secondary melting of materials, the occurrence of two melting peaks could be observed, which indicated the presence of two populations of crystallites in the crystal structure of PHB: large and small (unfinished). It should be noted that PHB materials with a Chl content of 1.25% had a low melting point during secondary melting, while with a Chl content of 1.5% this indicator was practically absent. The side effects can be explained by the strong intermolecular interaction between the polar groups of PHB and chlorophyll. Moreover, when melting the fibers, chlorophyll dissolves better in the PHB matrix, which leads to a significant inhibition of polymer crystallization. The effect is maximal at high concentrations of chlorophyll. These assumptions are largely consistent with a decrease in the melting temperature of the main PHB melting peak by more than 10 degrees at the first heating and by more than 15 degrees at the second heating. And at lower temperatures, smaller crystallites melt or have significant structural defects.

It is also possible to note a small peak at the first melting in the range of 60–80 °C, which is most pronounced for 0.5% chlorophyll. This peak probably signals the hydrogen bonds that are formed between Chl and PHB molecules. With an increase in the concentration of Chl, the molecules begin to aggregate to a greater extent, as a result of which the peak gradually disappears. This assumption is consistent with the peculiarities of the formation of defects on the fiber surface, confirming the difference in the intensity of the aggregation of chlorophyll molecules at low concentrations.

**Figure 5:**
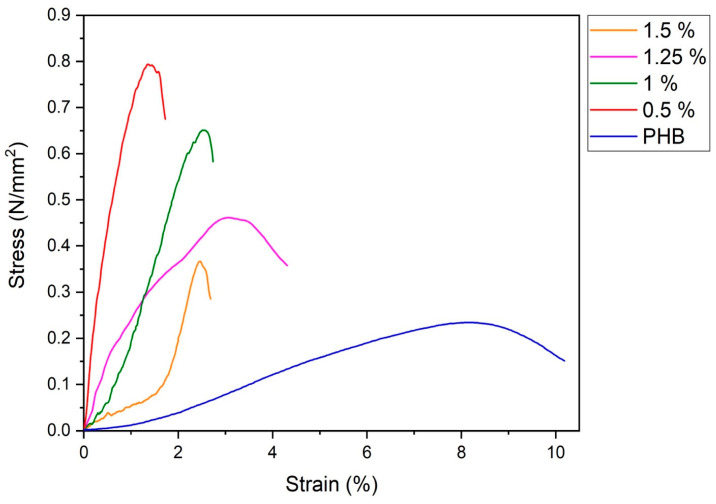
Mechanical properties of PHB-Chl electrospun materials (Δ ± 0.02 MPa).

The state of the internal structure of an electrospun fibrous material affects its strength properties. Figure 5 shows the dependence of the tensile stress on the deformation of nonwoven fibrous materials with different Chl contents.

All stress–strain curves of PHB-Chl electrospun materials have a similar appearance [60,61]. With increasing load, the relative elongation of nonwovens increases to the limit value, at which point the filaments rupture and the breaking load decreases until the complete destruction of the sample. As can be seen in Figure 5, the initial PHB material is characterized by a smooth increase in tensile stress from deformation. At the same time, it is characterized by an extremely low value of the breaking stress at rupture relative to compositions with Chl. When chlorophyll is introduced into PHB, there is a decrease in elongation and a significant increase in the tensile strength of nonwoven fibrous materials. When chlorophyll is added to PHB, fibrous materials become more rigid and brittle, as was previously observed for some Chl derivatives [62]. This may be caused by a significant intermolecular interaction, which leads to a decrease in the mobility of PHB macromolecules in amorphous regions.

As shown in Figure 5, the strength of PHB-Chl fibrous materials decreases with increasing Chl concentration. This strength behavior can be explained by a decrease in the degree of crystallinity and the size of crystallites (Figure 4, Table 3) and an increase in fiber defects due to the agglomeration of Chl molecules (Figure 1). With a Chl content of 0.5%, the smallest defects can be observed on the fibers and, accordingly, they are characterized by the highest strength values.

**Figure 6:**
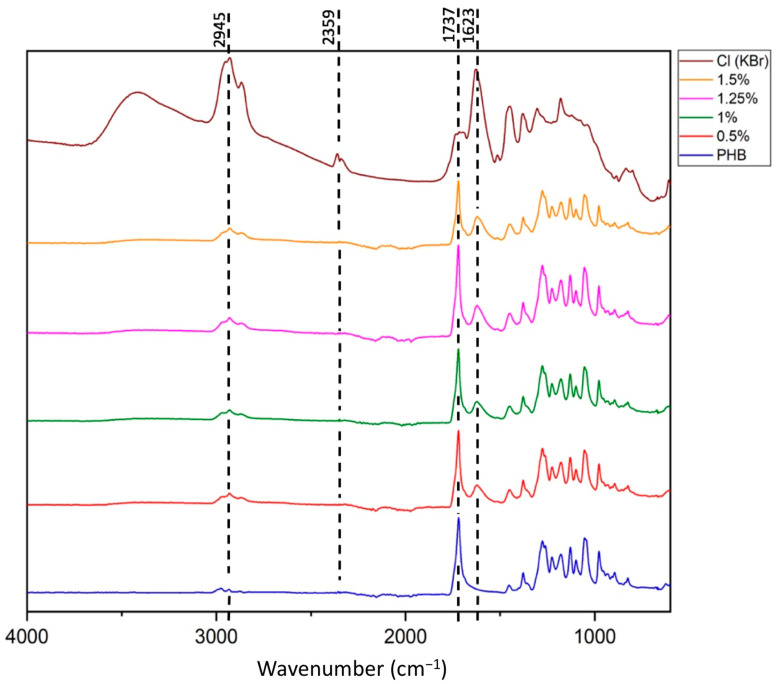
FTIR spectra of pure Chl and PHB-Chl electrospun materials (incomplete internal reflection mode).

Since Chl has a high bactericidal effect, antibacterial properties should be expected in nonwoven fibrous matrices of PHB-Chl. The bactericidal effect may occur in the case of chlorophyll release on the surface of the fibers. To establish this fact, the surface of the fibers was investigated using FTIR spectroscopy methods (incomplete internal reflection mode) and by measuring the wetting edge angle. The FTIR spectra are shown in Figure 6.

The analysis of the FTIR spectra (Figure 6) showed that the most pronounced chemical groups of PHB correspond to peaks at 1721 cm^−1^ (group C=O), 1052 cm^−1^ (group C-O-C), 1278 cm^−1^ (group -CH_3_) and 3000-2700 cm^−1^ (-CH fluctuations in the main chain). As can be seen, all the characteristics of the signals of the pure PHB are found in the composite materials. In addition, a new peak was detected in the area of 1640 cm^−1^, which is characteristic for –NH groups. This peak is clearly visible on the spectrum of pure Chl. On the spectra of fibrous materials, the superposition of the Chl peak on the corresponding peaks of PHB in the region of 2900–3000 cm^−1^ is clearly noticeable, leading to the formation of a triplet. The new peak observed at 3440 cm^−1^ corresponds to adsorbed water. However, this peak is pronounced in pure Chl and may contribute to the FTIR spectrum of PHB-Chl systems. In general, based on the results of the analysis of the spectra of PHB-Chl fibrous materials, it can be concluded that chlorophyll is partially located on the surface of the fibers, which significantly affects the FTIR signal. The signal is taken from the surface (depth of penetration of the IR beam into the sample—approximately 2 μm, which is comparable to the average fiber diameter) and does not give a complete picture of the structural organization in the mass of fibers. In a large number of reports about PHB-based composites investigated by FTIR, it is noted that FTIR research is mainly of a qualitative nature [63,64]. It is also impossible to deny the existence of an intermolecular interaction between chlorophyll and PHB.

The results of measuring the wetting contact angle are presented in Table 4. A decrease in the wetting edge angle in the area of 0.5–1% Chl content in the compositions indicates a decrease in energy on the surface of the sample, i.e., a decrease in energy on the surface of the fibers, which may indicate an increase in the polarity or hydrophilicity of the material. The increase in polarity can be justified by the presence of chlorophyll molecules in the surface layers of fibers. An increase in the wetting angle at a 1.25–1.5% Chl content may be due to the presence of multiple thickenings on the fibers, leading to an increase in surface roughness and distortion of the wetting edge angle.

**Figure 7:**
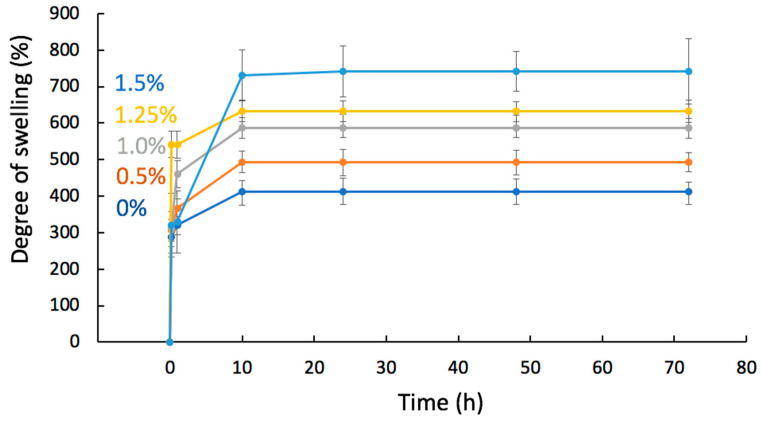
Swelling degree in PBS for PHB-Chl electrospun materials.

Since the wetting angle of electrospun materials could largely be determined by the surface morphology, both of individual fibers and of the entire system, the change in hydrophilicity was estimated by analyzing the water absorption of the material [65]. The swelling performance of an electrospun material largely depends on the degree of surface development and the proportion of open pores in the material, but the speed of the process and the slope of the swelling curve may indicate the hydrophobicity of the material [66]. The swelling degrees in PBS for the PHB-Chl electrospun materials are shown in Figure 7. It can be clearly seen that with an increase in the concentration of the additive, the percentage and amount of liquid that can be absorbed into the material increases linearly. Often, a more intense accumulation of liquid can be caused by hydrogen bonds that can form between the additive and water molecules [67]. Moreover, at concentrations of 0–1.25%, the same slope of the swelling curves and the same dynamics can be observed. And in the case of 1.5%, the process is more intensive, which can be explained by the high content of Chl. This experiment confirms that the contact angle of PHB in PHB-Chl electrospun materials is primarily determined by the surface structure.

### 3.2. Microbiological Testing of PHB-Chl Matrices

#### 3.2.1. MIC Determination

The antimicrobial activity of Chl, both when complexed with PHB and as a free form, has been investigated against the Gram-positive bacterium S. aureus strain 209-P. Both forms demonstrated antimicrobial activity across all tested concentrations.

After the first set of studies, the minimum inhibitory concentration (MIC) of Chl was found to be in a dose range from 11.25 to 15 μg. The MIC of Chl-PHB was in the range of 15–18.75 μg.

Following additional experiments, the MIC for the free form of Chl was determined to be 12.75 μg, while the MIC for the polymer form was 13 μg. After irradiation treatment, the MIC decreased to 10.25 μg for Chl and to 10 μg for Chl-PHB. Table 5 presents the data obtained.

Therefore, in order to achieve equivalent effects on microbial cells under irradiation conditions, 80% of the nonirradiated dose was sufficient for Chl and 76% was sufficient for Chl-PHB. 

Based on the data collected, a decision was made regarding the choice of preparation concentrations for further experimentation. In this process, both ineffective and excessively high concentrations were eliminated.

#### 3.2.2. Study of Microbial Growth Inhibition

MIC is not an absolute indicator but the lowest concentration of an antibacterial substance that causes suppression of microflora growth noticeable to the naked eye [68]. Therefore, it is necessary to determine the percentage of microorganism growth inhibition.

The spectrophotometric analysis of the microorganism cultures treated with Chl and Chl-PHB at the selected concentrations based on the MIC calculation demonstrated that the inhibition of Staphylococcus growth by these doses did not exceed 93%. Thus, the microorganisms were not completely killed, but their growth was slowed down or stopped. At the same time, slight growth (about 7%) persisted. When studying antimicrobial substances of various origins, researchers obtained the MIC_90_ values at which they noted the presence of 10% microorganism growth [69,70,71,72,73]. In other words, MIC was not a guarantee of bacterial destruction and did not provide complete control of the microbial population. According to J.M. Blondeau et al., to achieve a bactericidal effect, different MIC multiplicities may be required depending on the drug used, the type of microorganism and the density of the bacterial population [74].

In the current study, the inhibition exceeded 99.5% at an active substance concentration twice the MIC (Table 6). These results confirm a bactericidal or persistent bacteriostatic effect.

The data obtained allow us to hypothesize that in future experiments, optimal outcomes will be achieved with samples of the preparation containing an active substance concentration at least two times the MIC.

#### 3.2.3. Photodynamic and Light-Independent Inhibitory Effects

**Figure 8:**
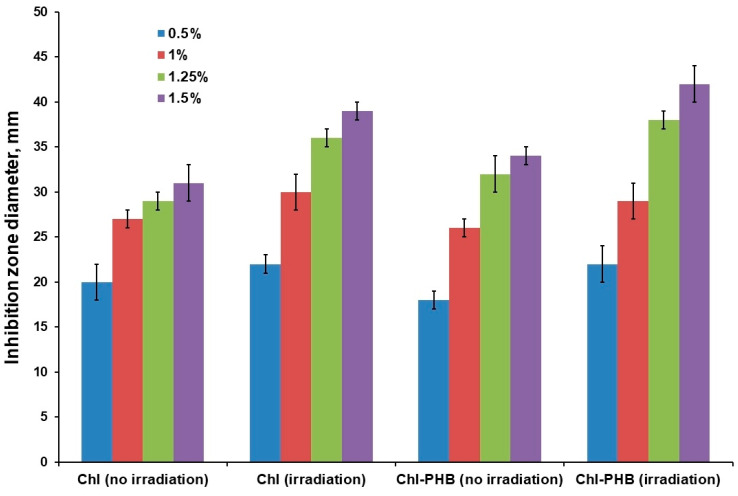
Inhibition of *Staphylococcus aureus* growth by Chl and Chl-PHB with irradiation exposure and without it.

The inhibitory effect study on a solid nutrient medium revealed a direct relationship between the diameter of the inhibition zone and the dose of active substance and irradiation exposure (Figure 8). Previously, the incorporation of Chl into the polymer matrix of poly(lactic acid) (PLA) was investigated. The Chl contents were 0.1–0.5 wt.% relative to the PLA mass. A content of 0.5% (15 µg) showed the best results [75]. Therefore, this was the starting point of the current study.

Figure 8 presents data on the diameter of the inhibition zone of growth on the fifth day of the experiment. This time period was chosen because the diameter of the zone at lower Chl concentrations (0.5% and 1%) decreased significantly over time, which may be attributed to incomplete suppression of bacterial growth.

In Petri dishes with PHB samples without active substances, there were no differences in microbial growth compared to the control culture. This suggests that the polymer has no inhibitory effect on Staphylococcus. This has been previously confirmed in other studies [76].

As can be observed from the data presented in Figure 8, at a 0.5% concentration of the active ingredient in the Chl-PHB complex and Chl solutions, there was no significant difference in their effects on microorganisms. However, after irradiation, an increase in efficiency of 10% and 22%, respectively, was noted for Chl and Chl-PHB.

Similarly, the irradiation efficiency increased by 11.1% and 11.5% for the polymeric and free forms at a Chl content of 1%.

The polymer complex without irradiation treatment was 10% more effective than the active substance at a concentration of 1.25%. The efficiency increase upon irradiation was 24.14% and 18.75% for the free and polymeric forms, respectively.

The polymer form containing 1.5% Chl was 9.7% more effective than the free form. The irradiation increased the inhibition level by 25.8% and 23.5% for Chl and Chl-PHB, respectively.

Therefore, it can be inferred that, out of all the preparations studied, the Chl-PHB complex containing 1.5% of the active substance demonstrated the highest antimicrobial activity, both when subjected to irradiation and in the absence thereof. Similar results were obtained with the polymeric form containing 1.25% of the active substance.

It should be noted that the dose-dependent effectiveness of the active substance on bacteria was demonstrated in our previous works devoted to the study of the antimicrobial properties of hemin in the composition of PHB, as well as Fe^III^Cl-Tetraphenylporphyrin in complex with poly-*N*-vinylpyrrolidone (PVP), against Gram-positive (*S. aureus*) and Gram-negative (*E. coli* and *S. typhimurium*) microorganisms. It was found that the higher the dosage of porphyrins, the more significant the inhibition of bacterial growth [74,76,77]. There are studies showing similar trends in the change in drug effectiveness not only depending on dosage, but also on the effect of irradiation. Thus, the work of K.A. Zhdanova et al. presents data proving an increase in the inhibitory effect of *meso*-aryl-substituted porphyrins and their complexes with Zn after irradiation [78]. I. Mendonça et al., studying the antimicrobial photodynamic potential of lipid extracts of microalgae (Bacillariophyta, Chlorophyta, Cyanobacteria, etc.), established the presence of a high inhibition degree of a *Staphylococcus aureus* methicillin-resistant strain as a result of irradiation [79]. This thesis was also confirmed in studies of phytochemicals (berberine, curcumin, farnesol, gallic acid and quercetin) by A.S.C. Gonçalves et al. [80], mesoporous silica nanoparticles loaded with IR780 iodide by H. Z. Alagha et al. [81], and natural anthraquinones—rubiadin 1-methyl ether—by J. Marioni et al. [82].

Summing up the obtained results, it should be noted that electrospun PHB-Chl systems obtained in the work differ from a wide range of analogues in terms of the very high productivity of the ES process, which does not require special additional synthesis conditions, and the simplicity of the system preparation in a single solvent. This was the first time such a significant effect of Chl on the supramolecular structure of PHB was recorded, where the Chl molecule prevents the crystallization of the polymer; and at a concentration of 1.5%, the proportion of the crystalline fraction of the polymer, as can be seen from the DSC results, decreased by more than three times. At the same time, despite the fragility of the obtained systems, their properties remain sufficient for use as materials with antimicrobial properties. Thus, the obtained materials with a highly developed structure are of interest for further research.

## 4. Conclusions

In this work, new nonwoven fibrous materials based on a natural biopolymer—poly-3-hydroxybutyrate—and chlorophyll with a pronounced antibacterial effect were obtained by electrostatic molding. The materials are characterized by heterogeneity with a chlorophyll content in the range of 0.5–1.5. The average diameter of the fibers is in the range of 1.2–6 microns. It has been established that the formation of the supramolecular structure of PHB fibers is strongly influenced by the intermolecular interaction between PHB and chlorophyll. At the same time, there is a significant slowdown in the rate of crystallization of PHB, which leads to a decrease in heat and melting point. As follows from the results of the IR spectroscopy study conducted in the mode of incomplete internal reflection and with a marginal wetting angle, chlorophyll molecules were detected in the surface layers of the PHB fibers, and thus the antibacterial effect of the obtained materials was justified. In microbiological experiments, MICs were determined for the free and polymer forms of Chl with irradiation and without it (12.75, 13.0, 10.25 and 10.0 μg, respectively). It was also found that the efficiency of bacterial cell growth suppression of more than 99% is achieved at a concentration of drugs no lower than 2×MIC. The Chl-PHB complex at active substance concentrations of 1.25 and 1.5% had a more significant inhibitory effect than free Chl. The irradiation increased the antimicrobial potential of both preparation forms. Potentially, the results obtained can be used in the development of new drugs against microbial infections. However, a more informed conclusion can be drawn after in vivo testing.

## Figures and Tables

**Table 1:** Parameters of PHB-Chl solutions for electrospinning.

Solution Number	Chl Content, %	PHB Content in ES Solution, g	Chl Content in ES Solution, mg	Electrical Conductivity, µS/cm	Viscosity, Pa s	Flow Rate of ES Solution, mL/min
1	0	3.5	0	10	1.00	150
2	0.5	3.5	9.0	12	0.90	210
3	1	3.5	17.5	12	0.80	213
4	1.25	3.5	22.0	12	0.85	225
5	1.5	3.5	26.5	14	1.20	235

**Table 2:** Morphological properties of PHB-Chl electrospun materials.

Chl Content, %	Average Diameter, μm	Surface Density, g/cm^3^	Thickness, mm
0	2.7	0.0018	0.0584
0.5	2.9	0.0079	0.2224
1	2.1	0.0060	0.2156
1.25	2.6	0.0052	0.1368
1.5	3.2	0.0097	0.3524

**Table 3:** Thermal properties of PHB in PHB-Chl electrospun materials.

Chl Content, %	Melting Temperature, °C (1 Heating) ∆ ± 0.2 °C	Enthalpy of Melting, J/g (1 Heating) ∆ ± 0.5 J/g	Melting Temperature, °C (2 Heating) ∆ ± 0.2 °C	Enthalpy of Melting, J/g (2 Heating) ∆ ± 0.5 J/g
0	176.4	78.43	173.5	80.5
0.5	163.3	44.58	158.3	38.74
1	163.5	48.44	159.3	46.81
1.25	160.2	32.31	155.7	8.235
1.5	156	25.05	-	-

**Table 4:** Wettability of PHB in PHB-Chl electrospun materials.

Chl Content, %	Contact Angle, Degree
0	117 ± 0.46
0.5	111 ± 0.48
1.0	112 ± 0.51
1.25	124 ± 0.48
1.5	126 ± 0.52

**Table 5:** Determination of Chl and Chl-PHB MICs relative to *S. aureus*.

Preparations	MIC, µg	
	No Irradiation	Irradiation (450 nm)
Chl	12.75 ± 0.5	10.25 ± 0.25
Chl-PHB	13.0 ± 0.5	10.0 ± 0.5

**Table 6:** Preparation-induced reduction in CFU growth of *S. aureus*.

Preparations	Reduction in CFU Growth, %	
	MIC	2 × MIC
Chl	91.93 ± 0.43	99.68 ± 0.1
Chl-PHB	92.67 ± 0.75	99.75 ± 0.1

## Data Availability

Data are contained within the article.
